# Omnichannel integration strategy based on BOPS

**DOI:** 10.1371/journal.pone.0293192

**Published:** 2023-12-21

**Authors:** Meirong Tan, Hao Li, Pei Yin, Hongwei Wang

**Affiliations:** 1 School of Economics and Management, Chongqing Jiaotong University, Chongqing, China; 2 School of Economics, Chongqing Finance and Economics College, Chongqing, China; 3 Business School, University of Shanghai for Science and Technology, Shanghai, China; 4 School of Economics and Management, Tongji University, Shanghai, China; Universidad Central de Chile, CHILE

## Abstract

Technological innovation and the upgrading of consumer preferences have greatly accelerated the rapid development of the "new retail" omnichannel model. Meeting the personalized and seamless interactive experience expected by consumers requires integrating the advantages of both offline and online channels and expanding the integrated and intelligent omnichannel layout. This has emerged as a complex problem that the industry urgently needs to address. In order to tackle this issue, we conducted a study on a Buy-Online-and-Pick-up-in-Store (BOPS) pricing game between offline stores and e-commerce departments, considering factors such as match probability and network cost of return. Furthermore, we proposed the Buy-Online-and-Pick-up-in-Store-and-Return-Online (BORO) strategy and conducted an analysis on the variation in market share and revenue levels for both offline stores and e-commerce departments under this strategy. The results demonstrate that: (i) the omnichannel strategy of BOPS can increase the revenues of both offline stores and e-commerce departments only when the distance costs are moderate; (ii) the BORO strategy provides greater benefits to offline stores compared to e-commerce departments; and (iii) the effectiveness of the BORO strategy is influenced by factors such as match probability, distance cost, and product return. This research not only provides a theoretical foundation but also practical insights for the strategic channel management of omnichannel brand merchants.

## Introduction

In recent years, the field of e-commerce has witnessed a continuous and rapid expansion. Market research firm eMarketer projects that global retail sales through e-commerce will reach a staggering $6.169 trillion by 2023. Online shopping has emerged as an indispensable method for consumers, offering convenience and accessibility. Among the global markets, China stands out with its substantial e-commerce industry surpassing that of other nations. Data from the Ministry of Commerce reveals that China’s online retail sales of physical goods exceeded 13 trillion yuan in 2022, experiencing a year-on-year increase of 4.0% [1]. Remarkably, China has maintained its position as the world’s largest e-commerce retail market for nine consecutive years. Nevertheless, the rapid growth of e-commerce has brought forth significant challenges, particularly in terms of product returns. For instance, in 2022, China witnessed a daily online shopping return rate of 10%, which escalated to 30% during the "double eleven" shopping festival. This high rate of returns poses a critical obstacle to the advancement of online retail.

On the other hand, the rapid development of e-commerce has had a significant impact on traditional brick-and-mortar stores, particularly those without inherent geographical advantages. Data from reliable sources reveals that nearly 10 million offline stores in China were forced to close in 2021. Nevertheless, the competitive advantages offered by traditional physical stores cannot be easily replaced by e-commerce counterparts. Notably, factors such as store design, layout, and ambiance can profoundly influence customer behavior by providing an immersive and engaging shopping experience [2, 3]. Furthermore, physical stores serve as venues where customers can derive instant gratification from their purchases [4]. As a result, there is a growing emphasis on the development of a new retail model that combines the strengths of both online and offline channels. According to a report by Forrester Research, approximately 80% of omnichannel retailers have embraced a Buy-Online-and-Pick-up-in-Store (BOPS) strategy, which has been implemented by prominent brands such as UNIQLO, Apple, Walmart, LIDS, The Children’s Place, Watsons, and many others.

However, the implementation of the BOPS strategy presents several challenges for retailers. When consumers place orders online, they are unable to directly experience the product or assess its physical attributes, such as the tactile sensation of clothing, accurate color perception, fit, and so on. This limitation hampers their ability to fully determine whether the ordered product suits their preferences. A higher product matching rate reduces the risk of returns for consumers, making them more inclined to make online purchases. Consequently, the conversion rate of BOPS channels may suffer. Conversely, if the product matching rate is low, consumers are more likely to choose the BOPS channel. However, when they visit the physical store to pick up the goods and find a mismatch with their expectations, the likelihood of canceling the order increases. This, in turn, raises the retailer’s operating costs and diminishes their profits.

Furthermore, the blind addition of new online channels by companies in the pursuit of mimicking the business model has created operational difficulties for offline stores. These challenges include inventory backlog, increased labor and rental costs, as well as high return rates on online platforms [5]. The intensified competition among channels has even eroded the benefits of the BOPS strategy. Consequently, some retailers, such as Clarks Outlet, H&M (China), and UNIQLO, have introduced the BORO policy [6]. The BORO approach offers potential benefits in terms of managing product returns and enhancing operational efficiency. For instance, BORO enables more consumers to pick up items from online stores at physical locations, thereby reducing the overall average return rate of products and mitigating the retailer’s return costs. Additionally, it can drive increased foot traffic in stores and spur in-store purchases, which can offset, and sometimes even surpass, the operating costs associated with the BOPS strategy. This explains why UNIQLO modified the return rules for BOPS channel products in offline stores across various regions in China and discontinued the option of returning online orders in physical stores.

The following questions hold significant practical importance within omnichannel retail: What are the variations in customer experience and behavior in BORO (Buy-Online-and-Pick-up-in-Store-and-Return-Online) operations? How does the implementation of BORO strategy impact the revenues of e-commerce departments and offline stores? Which offline stores are well-suited for adopting the BORO strategy? What factors pose limitations to the development of BORO? To address these inquiries, our study begins by examining the price game between offline stores and e-commerce departments, considering consumers’ utility derived from different channels. Our findings indicate that the BOPS mode increased profits for both subjects, but only when the distance cost is moderate. Building upon this, we proceed to analyze the channel integration strategy of BORO, which is based on the BOPS approach. Subsequently, we compare the differences in profits between the two strategies and observe that the BORO strategy offers greater advantages for offline stores as opposed to e-commerce departments. Finally, we conduct numerical studies to obtain further insights. Our results demonstrate that a lower return rate enhances the benefits of the BORO strategy for e-commerce departments. Additionally, implementing the BORO strategy for consumers residing relatively far away can boost the revenue of e-commerce departments. Conversely, for offline stores, the effectiveness of the BORO strategy diminishes as the match probability and distance cost increase. These findings underscore the importance for retailers to not only provide an integrated BORO service but also consider the strategic placement of offline stores. By elucidating the role of BORO in the context of offline stores and e-commerce departments, this study offers valuable insights for practitioners seeking to enhance their omnichannel return services.

## Literature review

In recent years, there has been a significant focus on the BOPS mode. Gallino and Moreno conducte a study utilizing transaction data from a dual-channel retailer to examine the impact of implementing a BOPS channel on its online and offline sales [7]. Gao and Su develope a NewsVendor model to explore the effects of adopting the BOPS strategy in traditional store operations [8]. Kim et al. employe a scenario-based factor survey method to empirically analyze the influence of product inventory availability information and pickup convenience on the development of the BOPS model [9]. In a similar vein, Jin et al. concentrate on the market with an omnichannel retailer and provided guidelines for determining the retailer’s optimal pricing and BOPS service area size [10]. Our research primarily aligns with two research streams, namely channel integration and omnichannel return.

The first research stream pertains to channel integration, which emphasizes the importance of achieving a high level of integration to enhance post-transaction performance in terms of speed, effort, and flexibility, while reducing customer anxiety and concerns [11, 12]. Within this stream, several studies have focused on analyzing the integration of the Buy-Online-and-Pick-up-in-Store (BOPS) strategy within omnichannel contexts. For instance, Zhao et al. focuse on the cancellation behaviour of orders caused by product mismatch and study the conditions for dual channel retailers to implement the (BOPS) strategy. The study found that when the product matching rate is high, and the operating cost of the BOPS channel is low, offline and online channels have the motivation to cooperate in implementing the dual channel BOPS strategy [13]. Jiang et al. investigate pricing and sales effort strategies within different integration models for BOPS sales encompassing both online and offline channels [14]. Fan et al. explore the influence of additional consumption, channel competition, and consumer channel behavior on pricing and service decisions [15]. Li et al. delve into manufacturer brand monopolies and retailer promotion strategies, analyzing how the design of prices and coupon face values can enhance brand competitiveness and revenue for manufacturers and retailers [16]. Shi et al. investigate the BOPS strategy with pre-orders and its impact on informed and uninformed consumers, identifying scenarios where the strategy proves beneficial based on thresholds for unit production cost and demand uncertainty [17]. Wang et al. constructe a game theory model to analyze the price and channel strategies of two retailers, considering disparities in shipping fees between their online channels [18].

The second research stream focuses on omnichannel returns, with several studies exploring the implications of the Buy-Online-and-Pick-up-in-Store-and-Return-in-Store (BORS) strategy on retailer pricing and revenue. For instance, Zhao et al. study the conditions for a dual channel retailers to implement an omnichannel model that combines BOPS strategy with cross channel return strategy(BORS) [19]. Yan et al. examine two competing dual-channel retailers and investigate the conditions under which either or both retailers should adopt the BORS strategy. Their findings indicate that the adoption of this strategy by both retailers significantly depends on the return rate and cross-selling profit [20]. Xie et al. investigate the impact of BORS channel integration on customer behavioral intentions, considering the mediating effect of customer satisfaction and the moderating effect of offline store characteristics [21]. Meanwhile, some studies do not focus on specific return methods. For example, Yang et al. propose a traditional dual-channel model that incorporates return risk and the BOPS strategy. They find that as the proportion of offline consumers with a high return risk increases, manufacturers are more likely to adopt BOPS. Conversely, when the proportion of offline consumers is low and the return risk is low, retailers are more inclined to adopt BOPS [22]. In a different vein, Zuo et al. discover that introducing BOPS in the context of returns can enhance consumers’ willingness to make offline purchases. However, they note that this may not always lead to a total increase in sales and that it may encourage retailers to prioritize sales in brick-and-mortar stores [23].

There have been limited studies focusing on the BORO strategy. Liu et al. examine online returns and channel costs, developing a profit model for brand retailers prior to implementing the BOPS channel. By considering consumer utility functions across the online, store, and BOPS channels, they find that opening the BORO channel can increase the total profit of brand retailers when the online return rate surpasses a certain threshold or when the BOPS channel is highly convenient [24]. Insufficient, they don’t delve into the consumer’s return decision and adopt the same price strategy for all channels. Nageswaran et al. study an omnichannel retailer’s pricing and return policy decisions by modelling prices to be identical across channels and allowing cross-channel returns. They find that firms with a significant store network and better in-store salvage opportunities might be better off charging a fee for BORO; otherwise, they offer full refunds [25].

Based on existing research, there have been numerous papers focusing on the BOPS omnichannel approach. However, only a subset of these papers considers consumer utility functions and profit models that incorporate match probability and network costs of return. In contrast to prior studies, the primary focus of this paper is the analysis of the BORO channel integration strategy for offline store and e-commerce department of a retailer. The objective is to examine the impact of the BORO strategy on the market share and profitability of offline store and e-commerce department. The key distinctions between this study and the most relevant papers are summarized in Table 1. In summary, this paper contributes to the existing literature in three significant ways. First, it establishes a price game between offline store and e-commerce department based on consumers’ utility across different channels and explores the equilibrium prices. Second, it analyzes the channel integration strategy of BORO in the context of BOPS. Lastly, it compares the differences in the two subjects’ profits under the two strategies and conducts numerical studies to provide valuable insights for retailers seeking to enhance their omnichannel retail operations.

**Table 1:** Differences between this research and the most relevant papers.

Articles	BOPS channel	Pricing	Return mode	Decision maker(s)
Zhao et al. (2022)	√	√	BORS	A dual-channel retailer
Yan et al. (2022)	√	√	BORS	Two dual-channel retailers
Yang et al. (2021)	√	√	—	A manufacturer and an offline retailer
Zuo et al. (2022)	√	√	—	A dual-channel retailer
Liu et al. (2019)	√	√	BORO	A dual-channel retailer
Nageswaran et al. (2020)	√	√	BORS, BORO	A dual-channel retailer
This paper	√	√	BORO	Offline store and e-commerce department of a retailer

## Model

We examine a dual-channel retailer whose online and offline channels belong to different management departments, and each department independently determines the corresponding retail price to attract different consumer segments. For instance, the online and offline channels of PEACEBIRD Men’s Wear Enterprise belong to different management departments, and each department independently decides the corresponding retail prices to achieve maximum revenue from the online and offline channels through differentiated pricing [13]. This dual-channel approach offers the advantage of leveraging the online channel to increase brand exposure and market coverage while providing enhanced experiences and services through the offline channel. However, it also introduces the challenge of potential conflicts and competition between the two channels, resulting in resource dispersion and inefficiency. In this study, we assume that an e-commerce department manages the online channel, denoted as "O", and a physical store manages the offline channel, denoted as "T". To maximize their profits, they have the option to cooperate or operate independently. In the cooperative scenario, consumers have three purchasing options: the offline channel(T channel), the online channel(O channel), and the Buy-Online-and-Pick-up-in-Store (BOPS) mode. Our model focuses on whether this type of retailer’s physical store and e-commerce departments are motivated to integrate online and offline channel cooperation to implement a BORO strategy and which physical stores suit the omnichannel online and offline channel integration model.

Prior to purchase, consumers are uncertain if the product will meet their specific needs. We assume that the product matches the consumer’s requirements with a probability of *ξ* and fails to match with a probability of 1 − *ξ*. Moreover, the probability of online and offline product matching is equal [19, 26]. The probability of matching is whether the product is suitable for consumers rather than whether consumers feel it is suitable after experiencing or recommending it. For example, whether the product’s size, colour, material and function match consumers’ needs. A customer has the option to visit the offline store, incurring a travel fee or distance cost denoted as *t* to physically examine a product before making a purchase. We assume that a consumer’s distance cost is uniformly distributed in [0, 1] [27, 28]. When there is a good match found in the offline store, consumers will make their purchases there. Otherwise, they may choose to buy from the online channel or opt for the BOPS mode. Making purchases from the online channel or through BOPS involves online transaction costs denoted as *h*, which represents customers’ willingness to wait for products to be shipped. Some customers are more willing to wait than others. We assume that *h* is independent of customers’ distance cost, which is still uniformly distributed in [0, 1]. Furthermore, we observe that after finding offline store matches, some shoppers choose to order online for a lower price and pick up their purchases in-store. These customers are aware of price variability across different channels and can avoid the hassle of online purchasing (e.g., waiting for delivery) by utilizing the online channel for ordering and in-store pickup. Alternatively, consumers can place their orders online and then physically visit the store for product pickup. Additionally, consumers can choose to purchase from the online channel and wait for the goods to be delivered.

Therefore, consumers derive different utilities from the three shopping modes. When consumers choose online channels, consumers have particular demand uncertainty due to the inability to truly touch and experience the product before purchasing. After receiving the product, they obtain the product value *r* by matching it with a probability of *ξ*. Therefore, the expected utility of consumers who choose online channels is *u*_*O*_ = *ξr* − *p*_*O*_ − *h*. When consumers choose offline channels, they only purchase when they have experienced the product and found that it matches their needs. That is, consumers who choose offline channels have a probability of *ξ* to purchase the matching product and obtain *r* − *p*_*T*_ − *t*, and a probability of 1 − *ξ* not to purchase the product and obtain −*t*. Therefore, the expected utility of consumers who choose to purchase products through offline channels is *u*_*T*_ = *ξ*(*r* − *p*_*T*_) − *t*. When consumers choose BOPS mode, they have to pay both online transaction and travel costs. The utility obtained through the BOPS channel is $u_{O}^{BS}=\xi \left(r-p_{O}-h\right)-t$.

However, when consumers choose the online channel or BOPS, there is a risk of product dissatisfaction due to the lack of "touch and experience" with the product. In this study, we initially assume that online orders cannot be returned but later relax this assumption. Under the BOPS model, we assume that the e-commerce department allocates a proportion of the revenue generated through the BOPS channel to the offline store, denoted by the ratio *σ*. The symbolic descriptions used in this paper are summarized in Table 2.

**Table 2:** Notation.

Notation	Description
*ξ*	Probability of a good match
*r*	Product utility in case of a good match
*t*	Distance cost to the offline store
*h*	Customers’ willingness to wait for products to be shipped
*p*_*i*_	Price of department *i*
*π*_*i*_	Department *i* profits
$\pi_{i}^{NB}$	Department *i* profits without BOPS
$\pi_{i}^{BS}$	Department *i* profits of BOPS
*σ*	Revenue distribution ratio of departments under the BOPS
*u*_*T*_	Expected surplus form T channel
*u*_*O*_	Expected surplus form O channel
$u_{O}^{R}$	Expected surplus form O channel under BORO strategy
$u_{O}^{BS}$	Expected surplus form BOPS channel under BORO strategy

In the basic model, we first consider the scenario without BOPS channel. A consumer can purchase from T obtains expected surplus *ξ*(*r* − *p*_*T*_) − *t*, or from O obtains expected surplus *ξr* − *p*_*O*_ − *h*. We assume that *r* is high enough that all consumers achieve a non-negative surplus in all strategies. A consumer will prefer T channel if *ξ*(*r* − *p*_*T*_) − *t* ≥ *ξr* − *p*_*O*_ − *h*. When *h* > *ξp*_*T*_ − *p*_*O*_ + *t*, the consumers in [0, *ξp*_*T*_ − *p*_*O*_ + *t*] will purchase from O channel and [*ξp*_*T*_ − *p*_*O*_ + *t*,1] will purchase from T channel. Therefore, the departments’ profit functions are $\pi_{T}^{NB}=\xi \left(1-\xi p_{T}+p_{O}-t\right)p_{T}$ and $\pi_{O}^{NB}=\left(\xi p_{T}-p_{O}+t\right)p_{O}$. Solving the first-order conditions(FOCs), we obtain the unique price equilibrium $p_{T}=\frac{2-t}{3\xi }$ and $p_{O}=\frac{1+t}{3}$. Two departments make profits are:

$$\pi_{O}^{NB}=\frac{{\left(1+t\right)}^{2}}{9}$$ (1)

$$\pi_{T}^{NB}=\frac{{\left(2-t\right)}^{2}}{9}$$ (2)

Under the BOPS strategy, a consumer has three options for purchasing: T channel, O channel, or BOPS channel. The utilities are as follows:

Under the BOPS strategy, a consumer has three options for purchasing: T channel, O channel, or BOPS channel. The utilities are as follows:

$$\left\{\begin{array}{l} u_{T}^{}=\xi \left(r-p_{T}^{}\right)-t\\ u_{O}^{}=\xi r-p_{O}-h\\ u_{O}^{BS}=\xi \left(r-p_{O}-h\right)-t \end{array}\right.\Rightarrow \left\{\begin{array}{l} u_{O}^{BS}>u_{O}^{}\Leftrightarrow h>\frac{t}{1-\xi }-p_{O}^{}\\ u_{O}^{BS}>u_{T}^{}\Leftrightarrow h<p_{T}^{}-p_{O}^{}\\ u_{O}^{}>u_{T}^{}\Leftrightarrow h<\xi p_{T}^{}-p_{O}^{}+t \end{array}\right.$$

**Fig 1:**
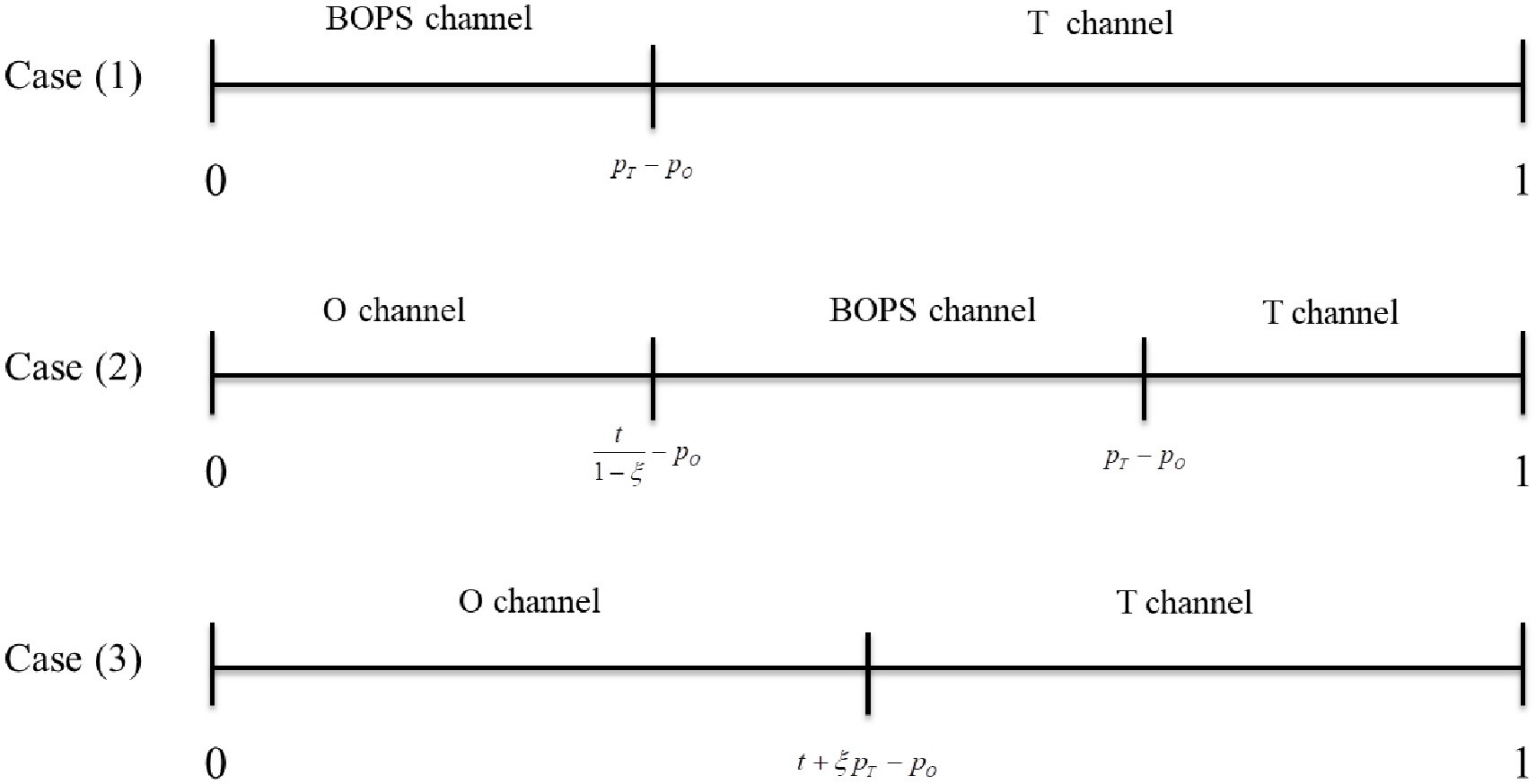
Equilibrium market segmentation.

If and only if $\frac{t}{1-\xi }-p_{O}<h<p_{T}-p_{O}$, the consumer *h* will choose the BOPS channel. There are three scenarios in the price game between two departments, as shown in Fig 1. Next, we discuss the price game.

Case (1): $p_{O}\geq \frac{t}{1-\xi }$

When $p_{O}\geq \frac{t}{1-\xi }$, $u_{O}^{BS}\geq u_{O}^{}$, i.e., the BOPS channel dominates the O channel. Therefore, the consumers in [0, *p*_*T*_ − *p*_*O*_) will choose the BOPS channel, and those in [*p*_*T*_ − *p*_*O*_, 1] will choose the T channel. The departments’ profit functions are $\pi_{O}^{BS}=\left(1-\sigma \right)\xi \left(p_{T}-p_{O}\right)p_{O}$ and *π*_*T*_ = *ξ*(1 − *p*_*T*_ + *p*_*O*_)*p*_*T*_ + *σξ*(*p*_*T*_ − *p*_*O*_)*p*_*O*_. When $t\leq \frac{1-\xi }{3-\sigma }$, the FOCs jointly lead to $p_{T}=\frac{2}{3-\sigma }$ and $p_{O}=\frac{1}{3-\sigma }$. Now departments make profits are:

$$\pi_{O}^{}=\pi_{O}^{BS}=\frac{\left(1-\sigma \right)\xi }{{\left(3-\sigma \right)}^{2}}$$ (3)

$$\pi_{T}^{}=\frac{\xi \left(4-\sigma \right)}{{\left(3-\sigma \right)}^{2}}$$ (4)

Then, the consumers in $\left[0,\frac{1}{3-\sigma }\right]$ will choose the BOPS channel, and the remaining consumer $\left[\frac{1}{3-\sigma },1\right]$ will choose the T channel. The proof process is provided in Appendix A.1 in S1 Appendix.

Case (2): $p_{T}\geq \frac{t}{1-\xi }\geq p_{O}$

In this case, we have $p_{T}-p_{O}\geq \xi p_{T}-p_{O}+t\geq \frac{t}{1-\xi }-p_{O}\geq 0$. The consumers in $\left[0,\frac{t}{1-\xi }-p_{O}\right)$ will choose the O channel, $\left[\frac{t}{1-\xi }-p_{O},p_{T}-p_{O}\right)$ consumers will choose the BOPS channel, and the other consumers [*p*_*T*_ − *p*_*O*_, 1] will choose the T channel. Now e-commerce department’s demand is $\frac{t}{1-\xi }-p_{O}^{}+\xi \left[p_{T}^{}-p_{O}^{}-\left(\frac{t}{1-\xi }-p_{O}^{}\right)\right]$. Two departments’ profit functions are:

$$\pi_{O}=\left(\frac{t}{1-\xi }-p_{O}^{}+\left(1-\sigma \right)\xi \left(p_{T}^{}-p_{O}^{}-\left(\frac{t}{1-\xi }-p_{O}^{}\right)\right)\right)p_{O}^{}$$ (5)

$$\pi_{T}^{}=\xi \left(1-p_{T}+p_{O}\right)p_{T}+\sigma \xi \left(p_{T}^{}-\frac{t}{1-\xi }\right)p_{O}^{}$$ (6)

Solving the first-order condition of Eqs (5) and (6). We obtain the equilibrium price are:

$$p_{O}^{}=\frac{\left(1-\xi -2t\right)\left(1-\sigma \right)\xi +2t}{\left(4-\xi \left(1-\sigma^{2}\right)\right)\left(1-\xi \right)}$$ (7)

$$p_{T}^{}=\frac{1}{2}+\frac{\left(1-\xi -2t\right)\left(1-\sigma^{2}\right)\xi +2t}{2\left(4-\xi \left(1-\sigma^{2}\right)\right)\left(1-\xi \right)}$$ (8)

When $\frac{\left(1-\xi \right)\xi }{\left(3+\sigma \right)\xi -2}\leq t\leq \frac{2\left(1-\xi \right)}{3}$, we have shown that these prices are equilibrium prices (see Appendix A.2 in S1 Appendix).

Case (3): $p_{T}\leq \frac{t}{1-\xi }$

When $p_{T}\leq \frac{t}{1-\xi }$, we have *p*_*T*_ − *p*_*O*_ ≤ *ξp*_*T*_ − *p*_*O*_ + *t* ≤ *t* −(1 − *ξ*)*p*_*O*_. In this instance, the distance cost is so high that the BOPS channel does not arise. Those consumers in [0, *ξp*_*T*_ − *p*_*O*_ + *t*) choose the O channel, and the remaining consumers choose the T channel. Departments make profits are *π*_*T*_ = *ξ*(1 − *ξp*_*T*_ + *p*_*O*_ − *t*)*p*_*T*_ and *π*_*O*_ = (*ξp*_*T*_ − *p*_*O*_ + *t*)*p*_*O*_. The FOCs yield $p_{T}=\frac{2+t}{3\xi }$ and $p_{O}=\frac{1+t}{3}$. Now departments’ profits are:

$$\pi_{O}^{}=\frac{{\left(1+t\right)}^{2}}{9}$$ (9)

$$\pi_{T}^{}=\frac{{\left(2-t\right)}^{2}}{9}$$ (10)

When $\frac{2\left(1-\xi \right)}{1+2\xi }\leq t<1$, we can verify these prices are equilibrium. Neither department will deviate from any higher $\frac{t}{1-\xi }$ (see Appendix A.3 in S1 Appendix).

We have summarized the above analysis in Proposition 1.

**Proposition 1.** Under the three equilibriums, departments’ prices and profits at different distances are:

i. For low distance cost ($t\leq \frac{1-\xi }{3-\sigma }$), the unique equilibrium is $p_{O}=\frac{1}{3-\sigma }$ and $p_{T}=\frac{2}{3-\sigma }$, and consumers in $\left[0,\frac{1}{3-\sigma }\right]$ will choose the BOPS channel.
ii. For intermediate distance cost ($\frac{\left(1-\xi \right)\xi }{\left(3+\sigma \right)\xi -2}\leq t\leq \frac{2\left(1-\xi \right)}{3}$), the unique equilibrium is $p_{O}^{}=\frac{\left(1-\xi -2t\right)\left(1-\sigma \right)\xi +2t}{\left(4-\xi \left(1-\sigma^{2}\right)\right)\left(1-\xi \right)}$ and $p_{T}^{}=\frac{1}{2}+\frac{\left(1-\xi -2t\right)\left(1-\sigma^{2}\right)\xi +2t}{2\left(4-\xi \left(1-\sigma^{2}\right)\right)\left(1-\xi \right)}$. O’s profits increase, and T’s profits decrease in distance cost.
iii. For high distance cost($\frac{2\left(1-\xi \right)}{1+2\xi }\leq t<1$), the unique equilibrium is $p_{O}=\frac{1+t}{3}$ and $p_{T}=\frac{2-t}{3\xi }$. The BOPS channel does not arise in this case. Department O’s prices and profits increase with distance cost, while T’s prices and profits decrease.

**Fig 2:**
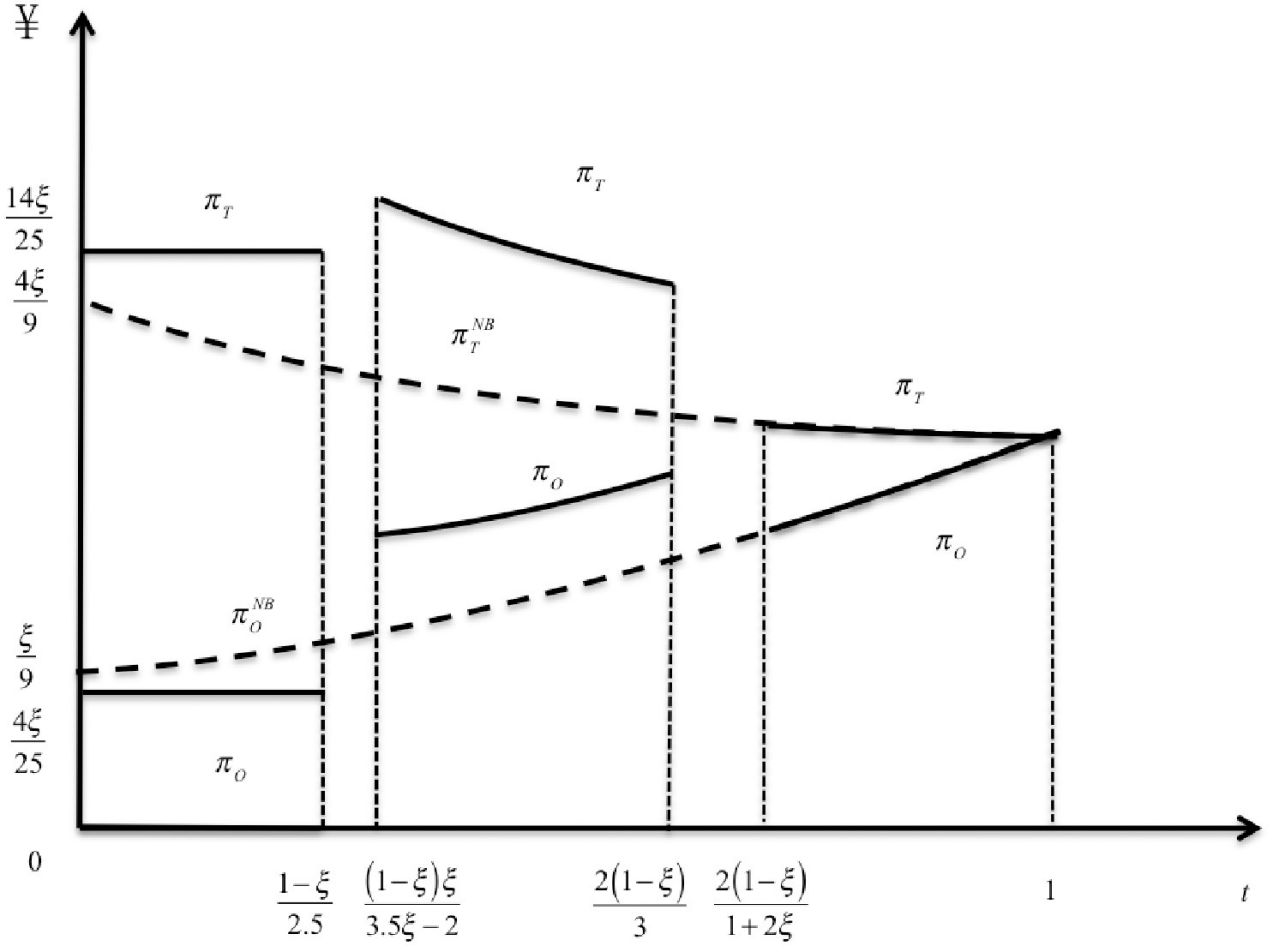
Retaiers profits as functions of distance cost.

Now, we discuss how departments’ profits vary with distance cost in three scenarios(see Fig 2, $\sigma =\frac{1}{2}$). In Case (1), the distance cost is low so that all consumers are willing to go to the offline store. Subsequently, consumers will compare the price and purchase from the lower, which will intensify competition. In this case, both departments’ prices are lower than those without BOPS. However, due to the existence of the distribution ratio of revenue of BOPS channel, O’s profits are lower, and T’s are higher than without BOPS. In Case (2), T’s demand function is the same as in Case (1). However, O’s demand elasticity increases from *∂D*_0_/*∂p*_*O*_ = *ξ* to 1, because the consumers with low distance cost choose the BOPS. This increases competition, resulting in higher prices and profits for each department compared to Case (1). In Case (3), the distance cost is so high that no consumer adopts the BOPS. O’s demand function remains the same as in Case (2), however, the demand elasticity of T decreases from |*∂D*_*T*_/*∂p*_*T*_ = *ξ* to *ξ*^2^|, which weakens price competition. Therefore, as the distance cost increases, the demand and profits for the offline store decrease, while the demand and profits for the e-commerce department increase. It is worth noting that numerous offline stores are situated in bustling commercial areas. To capture a larger market share, an increasing number of traditional stores are embracing the BOPS sales model. Based on pertinent surveys, it has been found that 64% of the top 500 retailers in Europe have already implemented the BOPS service.

Next, we analyze how the equilibrium prices vary with matching probability *ξ*. In Case (1), all consumers ultimately went to an offline store, so the price is independent of *ξ*. In Case (2), both departments’ price increase in *ξ* because a higher matching probability will increase the demand for the BOPS channel and relax competition. In Case (3), T’s price decreases in *ξ*. At this point, the distance cost is very high, and departments are trying to sell their products and may reduce the price of products with a higher probability of matching. E-commerce department attracts only uninformed buyers, and its price is independent of *ξ*.

**Proposition 2.** When the distance cost is low, the BOPS mode will reduce O’s profits and increase T’s profits. When the distance cost is moderate, the BOPS mode will increase the profits of both departments.

Proposition 2 can be derived by comparing departments’ profits with and without BOPS. The BOPS mode does not always increase departments’ profits. The BOPS mode may increase profits only when the distance cost is moderate. This conclusion is consistent with Li et al. [29]. Without BOPS channel, consumers have two options: buy from O channel or T channel. The BOPS mode provides a new option that reduces price competition. Formally, without BOPS channel T’s demand elasticity is $\left|\partial D_{T}^{NS}/\partial p_{T}\right|=\xi^{2}$. In Cases (1) and (2), BOPS increases T’s demand elasticity to *ξ*, thus increasing its profit. In Case (3), BOPS does not arise, the profit is the same as before, and the profit decreases with *t*. As noted above, the BOPS mode does not entirely improve T’s situation.

## BORO strategy

In this section, we make the assumption that consumers who are satisfied with their online purchases have the option to return or exchange the products within a seven-day period without providing any specific reason. The return transportation insurance is extensive, allowing consumers to receive a full refund. Currently, there are two main methods for returning BOPS products: (1) returning them to an offline store, and (2) returning them to the address provided by online customer service. However, it is important to note that many offline stores do not support returns for BOPS channel purchases. Huang and Jin have examined the adverse consequences that may arise from the Buy-Online-and-Pick-up-in-Store-and-Return-in-Store (BORS) strategy [30]. When retailers offer BORS, there is a potential increase in the average return rate for products as more consumers transition from physical stores to online shops, thereby leading to higher return costs for the retailer. As an example, UNIQLO, a prominent retailer, has modified the return policies for BOPS channel products in offline stores in several regions, no longer allowing online orders to be picked up in-store. Given the current circumstances, such return rules are likely to be adopted more widely. Therefore, in our analysis, we consider the second return method: when consumers pick up goods from an offline store, they have the opportunity to inspect them on the spot. If they are unsatisfied, they can choose not to pick up the goods and apply for a refund online. For consumers who need to return products acquired through the BOPS channel, they are required to return them to the address designated by the online customer service, which we refer to as the Buy-Online-and-Pick-up-in-Store-and-Return-Online(BORO) strategy. This strategy can increase the number of in-store visits and purchases while reducing operating costs. It also saves consumers time, as many online orders come with return shipping insurance, eliminating the need for additional shipping fees, and consumers can even opt for doorstep pickups. The e-commerce department incurs a cost of to process each return order, representing the average unit cost for return processing. In general, we assume that products purchased through offline stores will not be returned. Consequently, consumers’ utility is derived from the three channels:

$$\left\{\begin{array}{l} u_{O}^{R}=\xi \left(r-p_{O}\right)-h\\ u_{O}^{BS}=\xi \left(r-p_{O}-h\right)-t\\ u_{T}^{}=\xi \left(r-p_{T}^{}\right)-t \end{array}\right.\Rightarrow \left\{\begin{array}{l} h>\frac{t}{1-\xi }\Leftrightarrow u_{O}^{BS}>u_{O}^{R}\\ h<t+\xi \left(p_{T}-p_{O}\right)\Leftrightarrow u_{O}^{R}>u_{T}^{}\\ h<p_{T}-p_{O}\Leftrightarrow u_{O}^{R}>u_{T}^{} \end{array}\right.$$

Consumers choose the BOPS channel if and only if $\frac{t}{1-\xi }<h<p_{T}-p_{O}$. Recall that, consumers in $\frac{t}{1-\xi }-p_{O}<h<p_{T}-p_{O}$ choose the BOPS channel in the basic model. The returns policy by O curtails BOPS. Next, we discuss the retail price game in two scenarios.

Case (4): $\frac{t}{1-\xi }\leq p_{T}-p_{O}$

When $\frac{t}{1-\xi }\leq p_{T}-p_{O}$, we have $\frac{t}{1-\xi }\leq t+\xi \left(p_{T}-p_{O}\right)\leq p_{T}-p_{O}$, then those in $\left[0,\frac{t}{1-\xi }\right]$ consumers will choose O channel, and return the unsatisfactory product. So the expected revenue per unit product for e-commerce department is *ξp*_*O*_ − (1 − *ξ*)*κ*. Consumers in $\left[\frac{t}{1-\xi },p_{T}-p_{O}\right]$ will choose BOPS channel, and the remaining consumers will choose T channel. At this case, the departments’ profit founctions are:

$$\pi_{O}=\frac{t}{1-\xi }\left[\xi p_{O}-\left(1-\xi \right)\kappa \right]+\left(1-\sigma \right)\xi \left(p_{T}-p_{O}-\frac{t}{1-\xi }\right)p_{O}-\left(1-\xi \right)\kappa \left(p_{T}-p_{O}-\frac{t}{1-\xi }\right)p_{O}$$ (11)

$$\pi_{T}=\xi \left(1-p_{T}+p_{O}\right)p_{T}+\sigma \xi \left(p_{T}-p_{O}-\frac{t}{1-\xi }\right)p_{O}$$ (12)

The first item in Eq (11) represents O’s profit through O channel, the second item represents the profit of BOPS channel, and the third item represents the return cost of processing BOPS channel products.

Slove the first-order condition of Eqs (11) and (12). The equilibrium price is:

$$p_{O}=\frac{1}{3-\sigma }+\frac{2t}{\left(3-\sigma \right)\left(1-\xi \right)}\left(\frac{\xi }{\left(1-\sigma \right)\xi -\left(1-\xi \right)\kappa }-1\right)$$ (13)

$$p_{T}=\frac{1}{2}+\frac{\left(\sigma +1\right)}{2\left(3-\sigma \right)}+\frac{t\left(\sigma +1\right)}{\left(3-\sigma \right)\left(1-\xi \right)}\left(\frac{\xi }{\left(1-\sigma \right)\xi -\left(1-\xi \right)\kappa }-1\right)$$ (14)

Where (1 − *σ*)*ξ* − (1 − *ξ*)*κ* ≠ 0. Then we get restrictive conditions $t\leq \frac{\left(1-\xi \right)\left(1-\sigma \right)\xi -{\left(1-\xi \right)}^{2}\kappa }{3\left(1-\sigma \right)\xi -2\left(1-\xi \right)\kappa }$, $\kappa <\frac{\left(1-\sigma \right)\xi }{1-\xi }$, $\sigma >\frac{2\xi -1}{\xi }$, $\xi >\frac{1}{2}$ and 3(1 − *σ*)*ξ* − 2(1 − *ξ*)*κ* ≠ 0. Obviously, neither department deviate from the equilibrium price. That is, T will not unilaterally deviate to any other price above $p_{T}-\frac{t}{1-\xi }$, and O will not deviate to any other price below $p_{O}+\frac{t}{1-\xi }$. The proof process is provided in Appendix A.4 in S1 Appendix.

Case (5): $\frac{t}{1-\xi }\geq p_{T}-p_{O}$

In this case, BOPS channel does not arise. Consumers in [0, *t* +*ξ*(*p*_*T*_ − *p*_*O*_)] will choose O channel, and return the unsatisfactory product. The remaining consumers will choose T channel. The departments’ profit functions are *π*_*O*_ = [*t* +*ξ*(*p*_*T*_ − *p*_*O*_)][*t* + *ξp*_*O*_ − (*1* − *ξ*)*κ*] and *π*_*T*_ = (1 − *t* − *ξ*(*p*_*T*_ − *p*_*O*_))*p*_*T*_. When $\frac{\left(1-\xi \right)-{\left(1-\xi \right)}^{2}\kappa }{2+\xi }\leq t<1$, the unique price equilibrium is $p_{T}=\frac{2-t+(1-\xi )\kappa }{3\xi }$ and $p_{O}=\frac{1+t+2\left(1-\xi \right)\kappa }{3\xi }$, and departments make profits:

$$\pi_{O}=\frac{{\left(1+t-\left(1-\xi \right)\kappa \right)}^{2}}{9}$$ (15)

$$\pi_{T}=\frac{{\left(2-t+\left(1-\xi \right)\kappa \right)}^{2}}{9}$$ (16)

Similarly, neither department will unilaterally deviate from the equilibrium price (see Appendix A.5 in S1 Appendix).

Proposition 3 summarizes the above analysis.

**Proposition 3.** Under the BORO strategy, when $t\leq \frac{\left(1-\xi \right)\left(1-\sigma \right)\xi -{\left(1-\xi \right)}^{2}\kappa }{3\left(1-\sigma \right)\xi -2\left(1-\xi \right)\kappa }$, $\kappa <\frac{\left(1-\sigma \right)\xi }{1-\xi }$, $\sigma >\frac{2\xi -1}{\xi }$, $\xi >\frac{1}{2}$, the equilibrium price is $p_{O}=\frac{1}{3-\sigma }+\frac{2t}{\left(3-\sigma \right)\left(1-\xi \right)}\left[\frac{\xi }{\left(1-\sigma \right)\xi -\left(1-\xi \right)\kappa }-1\right]$ and $p_{T}=\frac{1}{2}+\frac{\left(\sigma +1\right)}{2\left(3-\sigma \right)}+\frac{t\left(\sigma +1\right)}{\left(3-\sigma \right)\left(1-\xi \right)}\left[\frac{\xi }{\left(1-\sigma \right)\xi -\left(1-\xi \right)\kappa }-1\right]$; when $\frac{\left(1-\xi \right)-{\left(1-\xi \right)}^{2}\kappa }{2+\xi }\leq t<1$, the equilibrium price is $p_{T}=\frac{2-t+(1-\xi )\kappa }{3\xi }$ and $p_{O}=\frac{1+t+2\left(1-\xi \right)\kappa }{3\xi }$.

## Comparative analysis

We are going to compare the difference in profits before and after the implementation of the BORO strategy by the retailers. In Case (1), where the distance cost is low, consumers have two channels available for purchasing products: BOPS or the T channel. After implementing the BORO strategy, the total demand for online channel remains unchanged. However, it leads to an increase in total costs due to return costs, resulting in decreased profits. On the other hand, in this particular case, the BORO strategy has no impact on the profits of the T channel.

When the distance cost is medium (Case (2), Case (4)), we use $\pi_{O}^{R}$ to represent the profits of O under the BORO strategy and make $\Delta \pi_{O}^{}\text{=}\pi_{O}^{R}-\pi_{O}^{}$. Due to the inability to visually determine positive or negative, we assume that O receives the whole revenue from the BOPS channel to facilitate analysis. Making lim*σ* → 0, we have Δ*π*_*O*_ < 0, when $\frac{\left(1-\xi \right)\xi }{\left(3+\sigma \right)\xi -2}\leq t\leq \text{min}\left(\frac{2\left(1-\xi \right)}{3},\frac{\left(1-\xi \right)\left(1-\sigma \right)\xi -{\left(1-\xi \right)}^{2}\kappa }{3\left(1-\sigma \right)\xi -2\left(1-\xi \right)\kappa }\right)$, where $\xi >\frac{1}{2}$. The detailed proof process can be found in Appendix A.6 in S1 Appendix. However, it should be noted that the implementation of the BORO strategy has resulted in an increase in demand for the O channel. Nevertheless, the O must bear the return costs associated with both the O channel and BOPS channel. As a result, the revenue generated from the increased demand in the O channel is outweighed by the increased return costs, leading to a decrease in overall profit. This finding is in line with real-world observations. For instance, when UNIQLO, a prominent retailer, initially introduced the BOPS channel, it allowed returns in brick-and-mortar stores. This resulted in a substantial number of BOPS orders being returned to physical stores, thereby increasing the operating costs of those stores. Consequently, certain regions of UNIQLO have gradually discontinued the acceptance of BOPS returns in brick-and-mortar stores.

When the distance cost is medium, we use $\pi_{T}^{R}$ to represent the profits of T under the BORO strategy and make $\Delta \pi_{T}^{}\text{=}\pi_{T}^{R}-\pi_{T}^{}$. Similarly, we still assume that O gets all the revenue from BOPS channel, i.e., lim*σ* → 0. We find that $\Delta \pi_{T}^{}\text{=}\pi_{T}^{R}-\pi_{T}^{}>0$. That is to say, even if T does not participate in the revenue distribution of BOPS channel, implementing the BORO strategy can still improve T’s revenue.

When the distance cost is high (Case (5)), the BOPS channel does not exist. At this time, the two departments form a regional monopoly. They can use their advantages to set higher prices. Compared with Case (5), the O’s demand decreases, and we can see that the O’s demand elasticity decreased from |*∂D*_*O*_/*∂p*_*O*_ = 1 to *ξ*. Since it has to bear the cost of product returns on the O channel. As a result, the price increase cannot offset the cost of product returns, and the O’s will be hurt, which profits decrease from $\frac{{\left(1+t\right)}^{2}}{9}$ to $\frac{{\left(1+t-\left(1-\xi \right)\kappa \right)}^{2}}{9}$ because of the increase in product returns. On the other hand, when the distance cost is high (Case (5)), although the T’s demand elasticity remains unchanged, it benefits from the regional monopoly and its price rise, which leads to the profits increase from $\frac{{\left(2-t\right)}^{2}}{9}$ to $\frac{{\left(2-t+\left(1-\xi \right)\kappa \right)}^{2}}{9}$.

To sum up, Proposition 4 (Table 3) can be obtained, and the impact of the BORO strategy on T and O profits under different scenarios.

**Table 3:** The impact of BORO strategy on profit of T and O under different scenarios.

*t*	T’ profit	O’ profit
$t\leq \frac{1-\xi }{3-\sigma }$	—	↓
$\frac{\left(1-\xi \right)\xi }{\left(3+\sigma \right)\xi -2}\leq t\leq \text{min}\left(\frac{2\left(1-\xi \right)}{3},\frac{\left(1-\xi \right)\left(1-\sigma \right)\xi -{\left(1-\xi \right)}^{2}\kappa }{3\left(1-\sigma \right)\xi -2\left(1-\xi \right)\kappa }\right)$	↑	↓
$\text{max}\left(\frac{2\left(1-\xi \right)}{1+2\xi },\frac{\left(1-\xi \right)-{\left(1-\xi \right)}^{2}\kappa }{2+\xi }\right)\leq t<1$	↑	↓

Note: “↑”indicates increase; “↓”indicates reduction; “—”indicates that the system is not affected.

**Fig 3:**
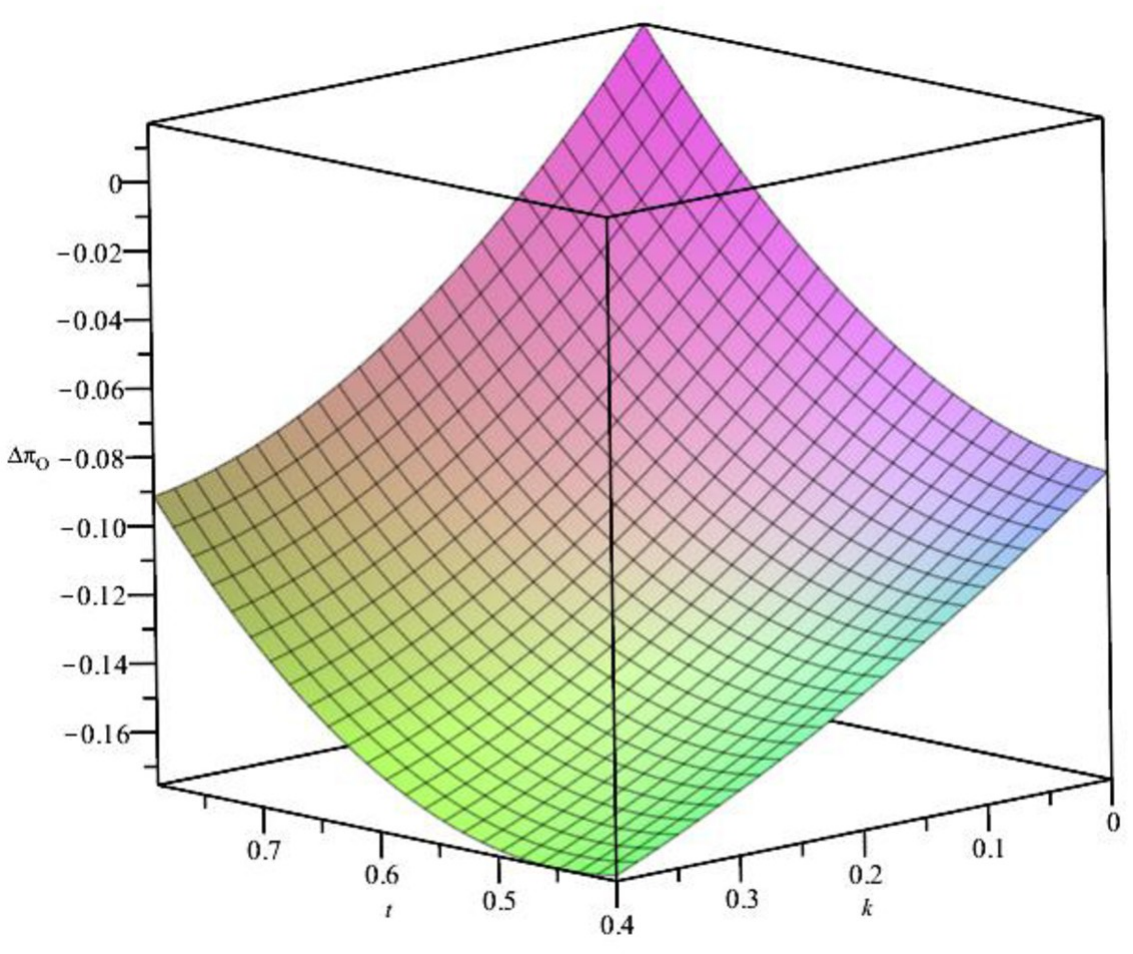
The relationship between *t*, *k* and Δ*π*_*O*_.

From the above analysis, the effectiveness of the BORO strategy is affected by many factors when the distance cost is medium. To verify its theoretical analysis, we assume $\xi \text{=}\frac{4}{5}$. The relationship of Δ*π*_*O*_, *t*, ***t***, and *k* is shown in Fig 3. From Fig 3, we can see $\Delta \pi_{O}=\pi_{O}^{R}-\pi_{O}^{}<0$ under the constraints of ***t*** and *k*, which is consistent with the previous theoretical analysis. Implementing the BORO strategy will reduce O’s revenue when the distance cost is moderate. In addition, the distance cost ***t*** is positively correlated with Δ*π*_*O*_. This means that implementing the BORO strategy for relatively far away consumers will increase O’s profits. It can also be seen from the figure that the rate of *k* return is negatively correlated with Δ*π*_*O*_. The lower the rate of return, the more favourable the BORO strategy is for O, which is also in line with reality.

**Fig 4:**
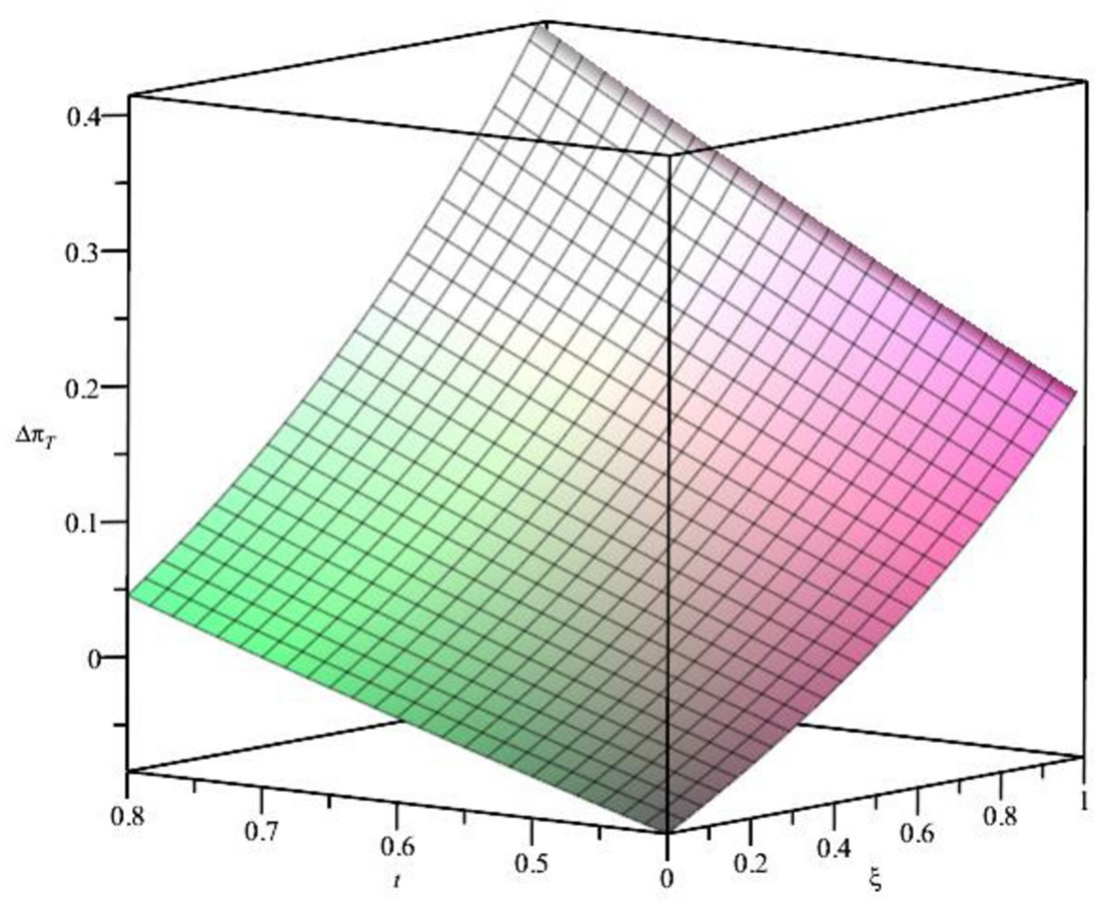
The relationship between *t*, *ξ* and Δ*π*_*T*_.

When the distance cost is moderate, the impact of the BORO strategy on T profit is shown in Fig 4. From Fig 4, we find that $△\pi_{T}=\pi_{T}^{R}-\pi_{T}^{}>0$ under the constraints of ***t*** and *ξ*, which verified the previous theoretical analysis, i.e., the BORO strategy can improve T’ profits. Moreover, there is a positive correlation between *ξ*, ***t*** and Δ*π*_*T*_, and with the increase of *ξ* and ***t***, the effectiveness of the BORO strategy is lower. Since the higher the match probability, the lower the return rate. With the increase in distance cost, more and more consumers choose the O channel, which leads to the decline of T’s demand and profits.

**Fig 5:**
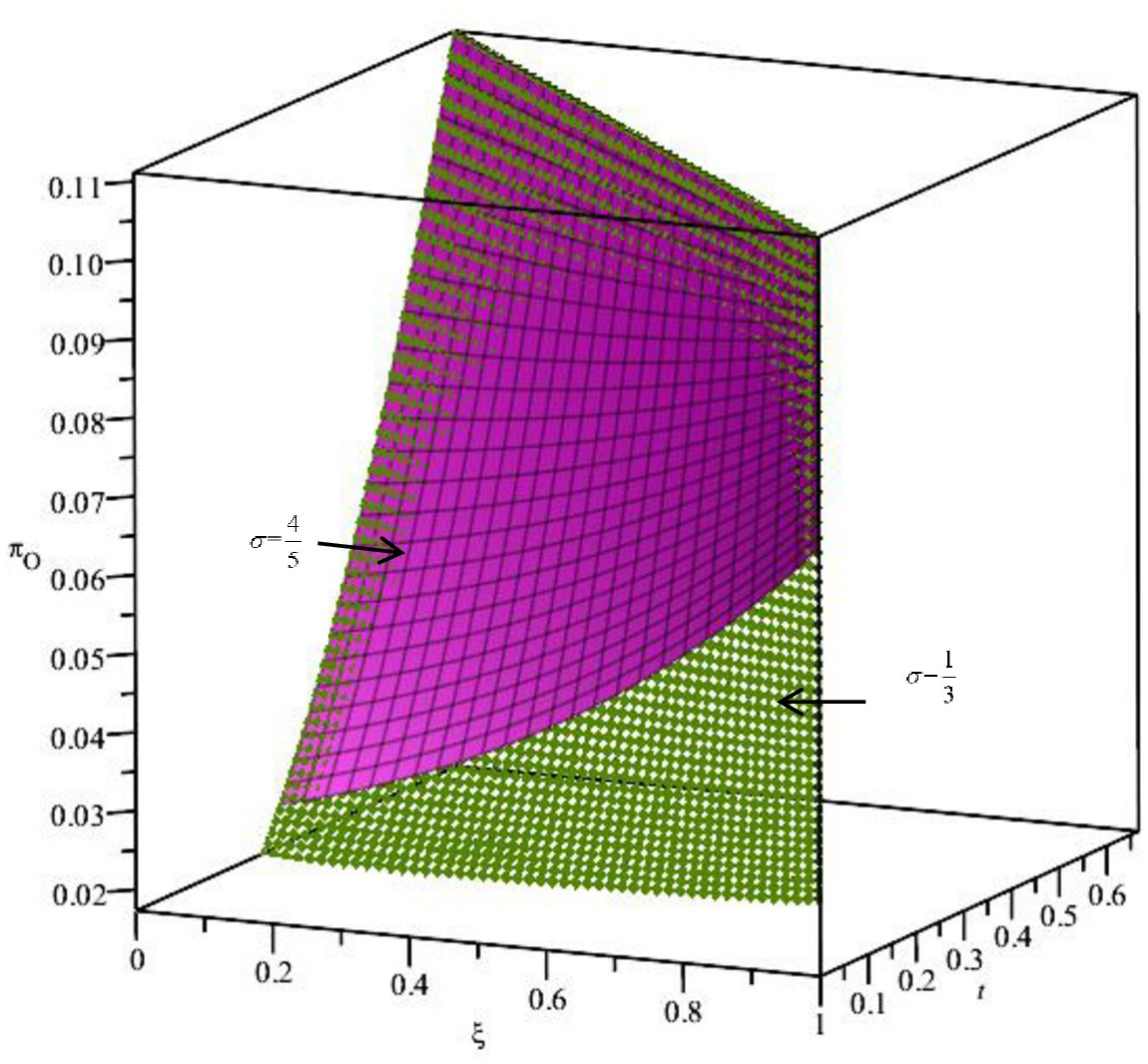
The relationship between *t*, *ξ* and *π*_*O*_.

**Fig 6:**
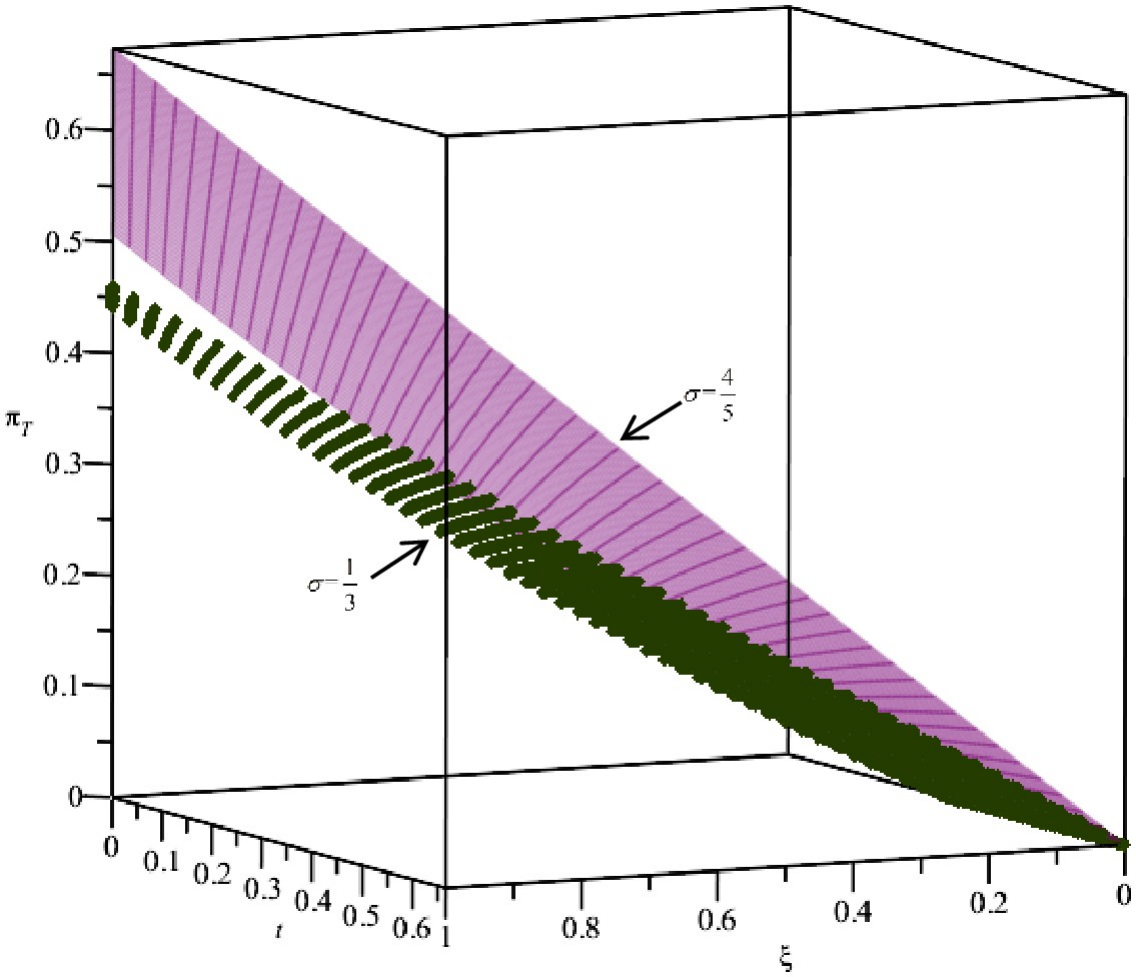
The relationship between *t*, *ξ* and *π*_*T*_.

In addition, when the distance cost is moderate, the profits of omnichannel retailers with the BORO strategy are mainly affected by ***t***, *ξ* and *σ*. To intuitively reflect their interrelationships, we assume $\sigma \text{=}\frac{1}{3}$ and $\sigma \text{=}\frac{4}{5}$, the relationships between *π*_*O*_, *π*_*T*_ and ***t***, *ξ* are shown in Figs 5 and 6, respectively. From Fig 5, we can see that *π*_*O*_ increases with *t* and *ξ*, and decreases with *σ*, while *π*_*T*_ decreases with *t* and increases with *ξ*, which is consistent with the theoretical analysis of Proposition 1. The relationship between *π*_*T*_ and ***t***, *ξ* in Figs 4 and 6 is consistent, indicating that the analysis of the BORO strategy is credible.

In practice, we have observed a significant number of brick-and-mortar store closures in China, leading to the shutdown of prominent shopping malls like BAIDA CBD in Hefei, Fuli Tianhui in Chengdu, and New World Department Store in Dalian, Shenyang, and Wuhan. These closures have been primarily driven by factors such as the rise of e-commerce, soaring rents, and the emergence of alternative shopping options, rendering brick-and-mortar stores less competitive in the market. However, it is worth noting that there are numerous other brick-and-mortar stores facing similar challenges. To address this situation, these stores can explore collaboration opportunities with e-commerce by serving as "pick-up stations," thereby expanding their physical presence and attracting in-store purchases.

For instance, UNIQLO which implemented the BORO policy has achieved remarkable popularity compared to other stores. We have also observed that many consumers visiting UNIQLO stores opt to pick up their online orders. In contrast, physical stores adjacent to UNIQLO that do not implement the BORO strategy have lower popularity, such as LILY, EIFINI, and URBAN REVIVO. Therefore, the BORO strategy effectively drives customer footfall to physical stores. Based on our analysis, physical stores situated at moderate to relatively long distances can partner with e-commerce to establish BOPS channels. For example, clothing and toy stores located near main roads, bus stops, or subway stations can attract consumers who conveniently pick up their orders while commuting to and from work.

The BORO strategy offers consumers the convenience of placing orders anytime and anywhere while enhancing order fulfillment flexibility through the option of picking up goods from nearby stores at convenient times. This approach helps avoid waiting times for express delivery and provides consumers with additional purchasing channels. From the perspective of physical stores, BORO generates customer foot traffic and reduces operating costs by eliminating the need to process online return orders. For online e-commerce, BORO reduces logistics costs and mitigates return rates. Consequently, this novel retail model integrates the strengths of both online and offline channels, leading to a Pareto improvement scenario.

## Conclusion

The integration of online and offline channels offers consumers a seamless shopping experience, particularly through the omnichannel retail model known as BOPS. This model enables consumers to enjoy real-time and personalized services while shopping online, eliminating the inconveniences associated with transportation and delivery and providing a smooth shopping journey. In this research, we concentrate on the price dynamics between an offline store and an e-commerce department in a context where competition and cooperation coexist.

To examine this dynamic, we develop a consumer utility function and a retailer profit model that take into account various factors, including the probability of product matching across different sales channels, the costs associated with brick-and-mortar shopping (such as distance costs), the challenges of online shopping (such as hassle costs), and the revenue distribution proportion within the BOPS channel. We then analyze the impact of the BOPS model on market demand, pricing decisions, and the profitability of both the offline store and the online e-commerce.

Furthermore, we propose a channel integration strategy called BORO, which combines the benefits of the BOPS model with cooperation between the offline and online departments. We assess the effects of implementing the BORO strategy on the revenues of both the offline and online departments. The research findings indicate the following key outcomes:

1. In comparison to dual-channel sales, when distance costs are low, the BOPS mode leads to a decrease in the e-commerce department’s revenue while increasing the revenue of the offline store. However, when the distance cost is moderate, the BOPS mode results in increased revenue for both departments.
2. The BORO strategy demonstrates greater advantages for offline stores than online e-commerce. Specifically, when distance costs are low, the BORO strategy leads to lower profits for the online e-commerce but does not significantly impact the revenue of offline stores. On the other hand, when the distance cost is moderate, although the BORO strategy increases the demand for the e-commerce department, it incurs return costs for both the O and BOPS channels. Consequently, the increase in revenue from the e-commerce department demand is outweighed by the increase in return costs, resulting in a decrease in total revenue for the e-commerce department. However, offline stores can leverage the benefits of the BORO strategy to achieve revenue growth. In situations where distance costs are high, the BOPS channel is not available, which reduces competition between the two departments, leading to higher prices and increased revenues for offline retailers. Nevertheless, for online e-commerce, the costs associated with returns contribute to a decrease in total revenue.
3. The effectiveness of the BORO strategy is influenced by factors such as match probability, distance cost, and product return rate. Particularly, in cases where distance costs are moderate, the lower the return rate, the more advantageous the BORO strategy becomes for the e-commerce department. Implementing the BORO strategy for consumers located at relatively greater distances can increase the revenues of online e-commerce. However, for offline stores, as match probability and distance costs increase, the effectiveness of the BORO strategy diminishes.

Our research findings offer valuable insights into the strategic management of omnichannel brand merchants. When it comes to offline shopping, consumers consider factors such as distance costs, timely and refined services, and the enjoyment of the shopping experience. Consequently, certain physical stores located at moderate to long distances can establish collaborations with e-commerce department to implement BORO channels. For example, clothing and toy stores situated near major roads, bus stops, or subway stations can adopt this model, focusing primarily on offline sales while leveraging the online channel for offline customer acquisition. Additionally, this model can be beneficial for retailers seeking rapid brand exposure and market expansion in the short term.

Consumers opt for online shopping primarily due to its convenience, affordability, and time-saving nature. However, they often face challenges such as low match probability and complex return processes. In response, e-commerce departments can utilize BORO strategies to enhance the quality of products and services, accurately match consumer supply and demand, and streamline return and exchange procedures. When integrating and optimizing retail channels, brand merchants should prioritize addressing consumers’ personalized and diverse needs. By combining the advantages of offline shopping pleasure and timely refined services with the online flow of information, capital, and logistics, they can develop an integrated and intelligent omnichannel framework.

## Limitations

There are several intriguing avenues for future research that arise from this study. Firstly, while this research examines a market with two entities, future studies could delve into the coexistence of multiple entities within the same market. Exploring the dynamics and strategies in such multi-player scenarios would provide valuable insights.

Secondly, in real-world scenarios, customers exhibit heterogeneity in their preferences for specific brands or services offered by brick-and-mortar stores and e-commerce departments. Understanding how retailers strategically navigate this heterogeneity and make choices to effectively cater to diverse customer segments would be an interesting area of investigation.

Lastly, this paper establishes a theoretical model that offers insights into the optimal pricing and channel strategies of competing retailers. However, to further validate and strengthen these findings, future research should incorporate more empirical analysis. By examining real-world data and conducting empirical studies, researchers can gain a deeper understanding of the practical implications and real-life outcomes of the proposed strategies.

Overall, exploring these areas of research would contribute to a comprehensive understanding of the complexities and dynamics involved in strategic pricing and channel management for retailers.

## Supporting information

S1 Appendix (DOCX)
