# Supplementary material for: Omnichannel integration strategy based on BOPS
Source: PLoS One. 2023 Dec 21;18(12):e0293192. doi: 10.1371/journal.pone.0293192 (PMC10734977; doi:10.1371/journal.pone.0293192)
Supplement: S1 Appendix — (DOCX) [file pone.0293192.s001.docx]

**Appendix**

**A.1**

Case (1)：$p_{O}\geq\frac{t}{1-\xi}$

Suppose O deviates to $p_{O}^{'}\leq\frac{t}{1-\xi}$, then consumers in $\left[0,\frac{t}{1-\xi}-p_{O}^{'}\right)$choose O channel and those in $\left[\frac{t}{1-\xi}-p_{O}^{'},p{}_{T}-p_{O}^{'}\right)$ choose BOPS channel and the remaining consumers $\left[p{}_{T}-p_{O}^{'},1\right]$ choose T channel $p_{T}=\frac{2}{3-\sigma}$. The O’s demand is $\frac{t}{1-\xi}-p_{O}^{'}+\xi\left[p_{T}-p_{O}^{'}-\left(\frac{t}{1-\xi}-p_{O}^{'}\right)\right]$and its profits are $\pi_{O}^{'}=p_{O}^{'}\left\{\frac{t}{1-\xi}-p_{O}^{'}+\left(1-\sigma\right)\xi\left(p_{T}-\frac{t}{1-\xi}\right)\right\}$. When $t<\frac{2\left(1-\xi\right)}{3-\sigma}$, O’s optimal deviating pricing is $p_{O}^{'}=\frac{t}{1-\xi}$, make profits $\pi_{O}^{'}=\frac{\xi t\left(1-\sigma\right)\left[2\left(1-\xi\right)-\left(3-\sigma\right)t\right]}{\left(3-\sigma\right)\left(1-\xi\right)^{2}}$.

Let $\Delta\text{=}\pi_{O}-\pi_{O}^{'}=\frac{\left(1-\sigma\right)\xi}{\left(3-\sigma\right)^{2}}-\frac{\xi t\left(1-\sigma\right)\left(2\left(1-\xi\right)-\left(3-\sigma\right)t\right)}{\left(3-\sigma\right)\left(1-\xi\right)^{2}}$

$$=\frac{\left(1-\sigma\right)\xi\left(1-\xi\right)^{2}-\left(1-\sigma\right)t\xi\left(2\left(1-\xi\right)-\left(3-\sigma\right)t\right)\left(3-\sigma\right)}{\left(3-\sigma\right)^{2}\left(1-\xi\right)^{2}}$$

The numerator $\left(1-\sigma\right)\xi\left(1-\xi\right)^{2}-\left(1-\sigma\right)t\xi\left(2\left(1-\xi\right)-\left(3-\sigma\right)t\right)\left(3-\sigma\right)$

$$=\left(1-\sigma\right)\left(\xi\left(1-\xi\right)^{2}-t\xi\left(2\left(1-\xi\right)-\left(3-\sigma\right)t\right)\left(3-\sigma\right)\right)$$

$$=\left(1-\sigma\right)\left(\left(1-\xi\right)-\left(3-\sigma\right)t\right)^{2}$$

$$\geq0$$

Such a deviation is not profitable for O since $\pi_{O}^{'}\leq\pi_{O}$,therefore, O will not unilaterally deviate from the equilibrium price. Similarly, T’s optimal deviating price at or below $p_{T}^{'}=p_{O}=\frac{1}{3-\sigma}$, yielding profits $\pi_{T}^{'}=\frac{\xi}{3-\sigma}$. However, T will not deviate to from a price since $\frac{\xi}{3-\sigma}<\frac{\xi\left(4-\sigma\right)}{\left(3-\sigma\right)^{2}}$.

**A.2**

Case (2)：$p_{T}\geq\frac{t}{1-\xi}\geq p_{O}$

In this case, the equilibrium price are:

$$p_{O}=\frac{\left(1-\xi-2t\right)\left(1-\sigma\right)\xi+2t}{\left[4-\xi\left(1-\sigma^{2}\right)\right]\left(1-\xi\right)}$$

$$p_{T}=\frac{1}{2}+\frac{\left(1-\xi-2t\right)\left(1-\sigma^{2}\right)\xi+2t}{2\left[4-\xi\left(1-\sigma^{2}\right)\right]\left(1-\xi\right)}$$

where $\frac{\left(1-\xi\right)\xi}{\left(3+\sigma\right)\xi-2}\leq t\leq\frac{2\left(1-\xi\right)}{3}$.

Suppose T deviates the equilibrium price $p_{T}^{'}\leq\frac{t}{1-\xi}$ , we have $p_{T}^{'}-p_{O}\leq\xi p_{T}^{'}-p_{O}+t\leq\frac{t}{1-\xi}-p_{O}$. Those consumers in $\left[\xi p_{T}^{'}-p_{O}+t,1\right]$ will choose T channel. So T’s profits are $\pi_{T}^{'}=$ $\xi\left(1-\xi p_{T}^{'}-p_{O}+t\right)p_{T}^{'}+\sigma\xi\left(p_{T}^{'}-\frac{t}{1-\xi}\right)p_{O}$ with $p_{O}=\frac{\left(1-\xi-2t\right)\left(1-\sigma\right)\xi+2t}{\left[4-\xi\left(1-\sigma^{2}\right)\right]\left(1-\xi\right)}$. The T’s optimal deviating price at $p_{T}^{'}=\frac{t}{1-\xi}$. When $\frac{\left(1-\xi\right)\xi}{\left(3+\sigma\right)\xi-2}\leq t\leq\frac{2\left(1-\xi\right)}{3}$, $\pi_{T}^{'}<\pi_{T}$. The proof process is similar to case (1) and is omitted here. If O deviates the equilibrium price $p_{O}^{'}\geq\frac{t}{1-\xi}$, those consumers in $\left[0,p_{T}-p_{O}^{'}\right]$will choose O channel. E-commerce department O’s is $\pi_{O}^{'}=\xi\left(p_{T}-p_{O}^{'}\right)p_{O}^{'}$with$p_{T}=\frac{1}{2}+\frac{\left(1-\xi-2t\right)\left(1-\sigma^{2}\right)\xi+2t}{2\left[4-\xi\left(1-\sigma^{2}\right)\right]\left(1-\xi\right)}$ . The O’s optimal deviating price at $p_{O}^{'}=\frac{t}{1-\xi}$, then $\pi_{O}^{'}=\left(1-\sigma\right)\xi\left(\frac{1}{2}+\frac{\left(1-\xi-2t\right)\left(1-\sigma^{2}\right)\xi+2t}{2\left[4-\xi\left(1-\sigma^{2}\right)\right]\left(1-\xi\right)}-\frac{t}{1-\xi}\right)\frac{t}{1-\xi}$. When $\frac{\left(1-\xi\right)\xi}{\left(3+\sigma\right)\xi-2}\leq t\leq\frac{2\left(1-\xi\right)}{3}$, $\pi_{O}^{'}<\pi_{O}$. Therefore, neither department will unilaterally deviate from the equilibrium price.

**A.3**

Case (3)：$p_{T}\leq\frac{t}{1-\xi}$

When $p_{T}\leq\frac{t}{1-\xi}$, the unique equilibrium price isand $p_{O}=\frac{1+t}{3}$. when $\frac{2\left(1-\xi\right)}{1+2\xi}\leq t<1$, neither department will deviate from any higher $\frac{t}{1-\xi}$. Suppose O deviates to$p_{O}^{'}>\frac{t}{1-\xi}$, the O’s demand is zreo. Suppose T deviates to$p_{T}^{'}>\frac{t}{1-\xi}$, the consumers in $\left[p_{T}^{'}-p_{E},1\right]$ will choose T channel. T’s profits are $\pi_{T}=\xi\left(1-p_{T}^{'}+p_{O}\right)p_{T}^{'}$ with $p_{O}=\frac{1+t}{3}$. Departments make profits are $\pi_{T}=\frac{\left(2-t\right)^{2}}{9}$ and $\pi_{O}=\frac{\left(1+t\right)^{2}}{9}$. When $\frac{2\left(1-\xi\right)}{1+2\xi}\leq t<1$, the T’s optimal deviating price at $p_{T}^{'}=\frac{t}{1-\xi}$，and make profits $\pi_{T}^{'}=\frac{\xi t\left[4\left(1-\xi\right)-\left(2+\xi\right)t\right]}{9\left(1-\xi\right)^{2}}$. Let $\pi_{T}-\pi_{T}^{'}=\frac{\left(2-t\right)^{2}}{9}-\frac{\xi t\left[4\left(1-\xi\right)-\left(2+\xi\right)t\right]}{9\left(1-\xi\right)^{2}}$, we obtain $\left[t-2(1-\xi)\right]^{2}+2\xi^{2}t^{2}>0$. This means that T deviating from the equilibrium price will lead to lower profits, and therefore will not unilaterally deviate from the equilibrium price.

**A.4**

Case (4): $\frac{t}{1-\xi}\leq p_{T}-p_{O}$

When $\frac{t}{1-\xi}\leq p_{T}-p_{O}$, the equilibrium price are:$p_{O}=\frac{1}{3-\sigma}+\frac{2t}{\left(3-\sigma\right)\left(1-\xi\right)}\left[\frac{\xi}{\left(1-\sigma\right)\xi-\left(1-\xi\right)\kappa}-1\right]$ $p_{T}=\frac{1}{2}+\frac{\left(\sigma+1\right)}{2\left(3-\sigma\right)}+\frac{t\left(\sigma+1\right)}{\left(3-\sigma\right)\left(1-\xi\right)}\left[\frac{\xi}{\left(1-\sigma\right)\xi-\left(1-\xi\right)\kappa}-1\right]$

Suppose O deviates to$p_{O}^{'}\geq p_{T}-\frac{t}{1-\xi}$, where $p_{T}=\frac{1}{2}+\frac{\left(\sigma+1\right)}{2\left(3-\sigma\right)}+\frac{t\left(\sigma+1\right)}{\left(3-\sigma\right)\left(1-\xi\right)}\left[\frac{\xi}{\left(1-\sigma\right)\xi-\left(1-\xi\right)\kappa}-1\right]$. Consumers in $\left[0,t+\xi\left(p_{T}-p_{O}^{'}\right)\right]$ choose O channel, and return the unsatisfactory product, and remaining consumers choose T channel. Then O’profits are $\pi_{O}^{'}=\left[t+\xi\left(p_{T}-p_{O}^{'}\right)\right]\left[\xi p_{O}^{'}-\left(1-\xi\right)\kappa\right]$. However, $\pi_{O}^{'}<\pi_{O}$when $t\leq\frac{\left(1-\xi\right)\left(1-\sigma\right)\xi-\left(1-\xi\right)^{2}\kappa}{3\left(1-\sigma\right)\xi-2\left(1-\xi\right)\kappa}$, $\kappa<\frac{\left(1-\sigma\right)\xi}{1-\xi}$, $\sigma>\frac{2\xi-1}{\xi}$, $\xi>\frac{1}{2}$.

Suppose T deviates to $p_{T}^{'}\leq p_{O}+\frac{t}{1-\xi}$, where $p_{O}=\frac{1}{3-\sigma}+\frac{2t}{\left(3-\sigma\right)\left(1-\xi\right)}\left[\frac{\xi}{\left(1-\sigma\right)\xi-\left(1-\xi\right)\kappa}-1\right]$. Those in $\left[t+\xi\left(p_{T}^{'}-p_{O}\right),1\right]$ consumers choose T channel, and remaining consumers choose O channel. Then, the T’ profits are$\pi_{T}^{'}=\xi\left[1-t-\xi\left(p_{T}^{'}-p_{O}\right)\right]p_{T}^{'}$. Clearly, $\pi_{T}-\pi_{T}^{'}>0$ with the previous restrictions.

**A.5**

Case (5): $\frac{t}{1-\xi}\geq p_{T}-p_{O}$

When $\frac{\left(1-\xi\right)-\left(1-\xi\right)^{2}\kappa}{2+\xi}\leq t<1$, the unique price equilibrium is $p_{T}=\frac{2-t+(1-\xi)\kappa}{3\xi}$ and $p_{O}=\frac{1+t+2\left(1-\xi\right)\kappa}{3\xi}$, and departments make profits$\pi_{T}=\frac{\left[2-t+\left(1-\xi\right)\kappa\right]^{2}}{9}$ and$\pi_{O}=\frac{\left[1+t-\left(1-\xi\right)\kappa\right]^{2}}{9}$.

Similaritily , neither department will unilaterally deviate form the equilibrium price. Suppose T deviates to $p_{T}^{'}\geq p_{O}+\frac{t}{1-\xi}$, consumers in $\left[p_{T}^{'}-p_{O},1\right]$ choose T channel and its profit function is $\pi_{T}^{'}=\xi\left[1-p_{T}^{'}+p_{O}\right]p_{T}^{'}$, where $p_{O}=\frac{1+t+2\left(1-\xi\right)\kappa}{3\xi}$. The T’s optimal deviating price at$p_{T}^{'}=p_{O}+\frac{t}{1-\xi}$.

However,$\pi_{T}-\pi_{T}^{'}=\frac{\left[2-t+\left(1-\xi\right)\kappa\right]^{2}}{9}-\xi\left(1-\frac{t}{1-\xi}\right)\left[\frac{1+t+2(1-\xi)\kappa}{3\xi}+\frac{t}{1-\xi}\right]>0$ when $\frac{\left(1-\xi\right)-\left(1-\xi\right)^{2}\kappa}{2+\xi}\leq t<1$. On the other hand, suppose O deviates to $p_{T}^{'}\geq p_{O}+\frac{t}{1-\xi}$, those consumers in $\left[0,\frac{t}{1-\xi}-p_{O}^{'}\right]$ choose O channel, and consumers in $\left[\frac{t}{1-\xi}-p_{O}^{'},p_{T}-p_{O}^{'}\right]$choose BOPS channel. The O’s profit functiong is $\pi_{O}^{'}=\left[\frac{t}{1-\xi}-p_{O}^{'}\right]\left[\xi p_{O}^{'}-\left(1-\xi\right)\kappa\right]+\left[\left(1-\sigma\right)\xi-\left(1-\xi\right)\rho\right]\left(p_{T}-\frac{t}{1-\xi}\right)p_{O}^{'}$ where $p_{T}=\frac{2-t+\left(1-\xi\right)\kappa}{3\xi}$. The O’s optimal deviating price at$p_{O}^{'}=p_{T}-\frac{t}{1-\xi}$. However, $\pi_{O}-\pi_{O}^{'}>0$ under restricted conditions.

**A.6**

When the distance cost is medium (case (2), case (4)), we use $\pi_{O}^{R}$ to represent the profits of O under the BORO strategy, and make$\Delta\pi_{O}\text{=}\pi_{O}^{R}-\pi_{O}$. To facilitate analysis, we assume that O receives full revenue from the BOPS channel. Making$\lim\sigma\to0$, we have :

Equation (11)

$$\pi_{O}^{R}$$

$$=\frac{t}{1-\xi}\left[\xi p_{O}-\left(1-\xi\right)\kappa\right]+\left(1-\sigma\right)\xi\left(p_{T}-p_{O}-\frac{t}{1-\xi}\right)p_{O}-\left(1-\xi\right)\kappa\left(p_{T}-p_{O}-\frac{t}{1-\xi}\right)p_{O}$$

$$=\left(1-\frac{1}{3}-\frac{2t}{3\left(1-\xi\right)}\left[\frac{\xi}{\xi-\left(1-\xi\right)\kappa}-1\right]\right)\left\{\left[\xi-\left(1-\xi\right)\kappa\right]\frac{1}{2}\left\{\frac{1}{3}+\frac{2t}{3\left(1-\xi\right)}\left[\frac{\xi}{\xi-\left(1-\xi\right)\kappa}-1\right]\right\}-t\kappa\right\}$$

$$=\frac{1}{9}\left(1-\frac{t\kappa}{\xi-\left(1-\xi\right)\kappa}\right)\left[\xi-\left(1-\xi\right)\kappa-4t\kappa\right]$$

Equation (5)$\pi_{O}=\left(\frac{t}{1-\xi}-p_{O}+\left(1-\sigma\right)\xi\left(p_{T}-p_{O}-\left(\frac{t}{1-\xi}-p_{O}\right)\right)\right)p_{O}$

$$=\frac{6t+\xi\left(t+1+\frac{\xi}{2}\right)\left(\left(1-\xi-2t\right)\xi+2t\right)}{\left(4-\xi\right)^{2}\left(1-\xi\right)}$$

$$\Delta\pi_{O}=$$

$$\pi_{O}^{R}-\pi_{O}$$

$$=\frac{1}{9}\left(1-\frac{t\kappa}{\xi-\left(1-\xi\right)\kappa}\right)\left[\xi-\left(1-\xi\right)\kappa-4t\kappa\right]$$

$$-\frac{6t+\xi\left(t+1+\frac{\xi}{2}\right)\left(\left(1-\xi-2t\right)\xi+2t\right)}{\left(4-\xi\right)^{2}\left(1-\xi\right)}$$

When $\frac{\left(1-\xi\right)\xi}{\left(3+\sigma\right)\xi-2}\leq t\leq\min\left(\frac{2\left(1-\xi\right)}{3},\frac{\left(1-\xi\right)\left(1-\sigma\right)\xi-\left(1-\xi\right)^{2}\kappa}{3\left(1-\sigma\right)\xi-2\left(1-\xi\right)\kappa}\right)$, where$\xi>\frac{1}{2}$. Using Matlab 2022, we obtain $\Delta\pi_{O}<0$. Due to the complexity of the expression, it is not presented here.

When the distance cost is medium, we use $\pi_{T}^{R}$represnets the revenue of T under the BORO strategy. We still assume that O gets all the revenue of BOPS channel, i.e., $\lim\sigma\to0$. Then equation (6)-(12):

$$\Delta\pi_{T}\text{=}\pi_{T}^{R}-\pi_{T}=\frac{4}{9}-\left(\frac{1}{2}-\frac{\left(1-\xi-2t\right)\xi+2t}{2\left(4-\xi\right)\left(1-\xi\right)}+\frac{\left(1-\xi-2t\right)\xi+2t}{\left(4-\xi\right)\left(1-\xi\right)}\right)\left(\frac{1}{2}+\frac{\left(1-\xi-2t\right)\xi+2t}{2\left(4-\xi\right)\left(1-\xi\right)}\right)$$

$$=\frac{4}{9}-\left(\frac{1}{2}+\frac{\left(1-\xi-2t\right)\xi+2t}{2\left(4-\xi\right)\left(1-\xi\right)}\right)^{2}$$

$$=\left(\frac{2}{3}-\frac{1}{2}-\frac{\left(1-\xi-2t\right)\xi+2t}{2\left(4-\xi\right)\left(1-\xi\right)}+\right)\left(\frac{2}{3}+\frac{1}{2}+\frac{\left(1-\xi-2t\right)\xi+2t}{2\left(4-\xi\right)\left(1-\xi\right)}\right)$$

$$=\left(\frac{7}{6}+\frac{\left(1-\xi\right)\xi+2t\left(1-\xi\right)}{2\left(4-\xi\right)\left(1-\xi\right)}\right)\left(\frac{1}{6}-\frac{\left(1-\xi-2t\right)\xi+2t}{2\left(4-\xi\right)\left(1-\xi\right)}\right)$$

Simplifying $\frac{1}{6}-\frac{\left(1-\xi-2t\right)\xi+2t}{2\left(4-\xi\right)\left(1-\xi\right)}$ $=\frac{\left(4-\xi\right)\left(1-\xi\right)-3\left(1-\xi-2t\right)\xi+6t}{6\left(4-\xi\right)\left(1-\xi\right)}$, when$\left(4-\xi\right)\left(1-\xi\right)-3\left(1-\xi-2t\right)\xi+6t>0$, i.e., $t>\frac{2\left(1-\xi\right)}{3}$, $\pi_{T}^{R}-\pi_{T}>0$. However, in case (2), we have $\frac{\left(1-\xi\right)\xi}{\left(3+\sigma\right)\xi-2}\leq t\leq\frac{2\left(1-\xi\right)}{3}$, thus $\pi_{T}^{R}-\pi_{T}>0$.
